# Development and Evaluation of the POPBL (Patient-Oriented Problem-Based Learning) Module in Pathology: A Comparative Analysis of Performance and Perception Among Second-Year Pathology Students

**DOI:** 10.7759/cureus.28885

**Published:** 2022-09-07

**Authors:** Killol N Desai, Vidya K Satapara, Gunvanti B Rathod, Alpeshkumar M Maru

**Affiliations:** 1 Pathology, Nootan Medical College and Research Centre, Visnagar, IND; 2 Anatomy, Nootan Medical College and Research Centre, Visnagar, IND; 3 Pathology, All India Institute of Medical Sciences, Bibinagar, Hyderabad, IND; 4 Pathology, Dr. N.D. Desai Faculty of Medical Science and Research, Nadiad, IND

**Keywords:** curriculum implementation support programme (cisp), demonstration -observation - assistance - performance (doap), competency based medical education (cbme), students centered, deca and penta head microscope, popbl (patient oriented problem based learning)

## Abstract

Background and objective

Employing the POPBL (Patient-Oriented Problem-Based Learning) method to teach students offers a fresh take on the classroom experience. It helps to enhance the motivation of the students, improves knowledge, self-learning behavior, and clinical reasoning, and also helps to promote long-lasting memory. In our medical college, we adopted a newer technology-oriented method with the use of case history, laboratory findings, a gross specimen of the same case, microscopic live sessions via Deca and Penta head microscopes, television, and microscopic camera. In light of this, in this study, we aimed to develop a patient- and technology-oriented new Problem-Based Learning (PBL) method and compare its effectiveness with the traditional tutorial method.

Materials and methods

A total of 149 second-year MBBS students were enrolled in the study. Consent was taken from all students. A total of eight systems of systemic pathology from the second-year MBBS curriculum were selected. Of the eight systems, four were covered under POPBL with gross and microscopic features associated with the help of newer-generation audiovisual aids, and the other four systems were covered under the traditional tutorial/lecture method. The evaluation was performed using prevalidated objective types of questions after exposure of about one week. The objective was to evaluate and compare the outcomes and students' performance between these two sets of pathology systems.

Results

Students gave excellent responses. Performance (87.92% of students had scores >75%) and attendance (94.14%) parameters with respect to POPBL gross and microscopic features associated with the help of newer-generation audiovisual aids like Deca and Penta head microscopes were superior compared to the traditional tutorial/lecture method, where 53.02% of students scored more than 75% and the attendance was 76.12%. The difference in attendance was also statistically significant (p=0.05).

Conclusion

Using POPBL instead of standard tutorial/lecture methods leads to better outcomes. Students also found POPBL more appealing than standard lectures. It is a student-centered method that provides a significant level of motivation and encourages active participation among students. The efficacy of this new way of teaching and demonstrating will attract more students to this method.

## Introduction

Several novel teaching and learning methods have been introduced in medical education recently [1]. Medical education's primary purpose is to develop students into capable and competent doctors. We would want to find out whether, as Melinkof said, we are unwell. With the assistance of cutting-edge audiovisual tools, our school is implementing a method called POPBL (Patient-Oriented Problem-Based Learning). POPBL is a revolutionary teaching and learning system that fosters long-term memory formation, increases intrinsic motivation, facilitates self-directed learning, and improves clinical reasoning. In 1899, 100 years after the advent of medical education, Sir William Osler observed that the complexity of medical education had already exceeded instructors' abilities. This necessitated the need to adopt a new strategy to better fulfill the educational demands of pupils [2]. In 1960, the McMaster University Medical School in Canada established and developed Problem-Based Learning (PBL) [3,4]. PBL has long been the technique of choice in many American medical schools [5]. The National Medical Council (NMC) in India has recommended that each medical institution use the PBL technique in various ways. Medical education is increasingly being conducted in accordance with the principles of competency-based medical education (CBME). In terms of medical education program assessment and evaluation, it has been described as a strategy focused on outcomes [6,7]. The major characteristics of the Curriculum Implementation Support Programme (CISP) are its longitudinal nature, provision of development of feedback, and authentic settings. Various Asian countries like Nepal, China, Singapore, Hong Kong, and Malaysia have also implemented the PBL method, but they are facing some problems similar to Indian medical colleges [6-10]. POPBL involves patient-oriented research and focusing on the problems associated with them and then analyzing the problems and resolving them, and all of these have to be executed in a timely manner. POPBL and PBL are similar to each other; the only difference lies in the fact that POPBL is used for patient orientation and long-term continued analysis, whereas the term PBL is associated with short-term goals. POPBL needs to be implemented to achieve a greater understanding of the clinical aspects and for regular follow-up of the patients.

In our medical college, we are adopting newer technology-oriented methods with the use of case history, laboratory findings, gross specimens of the same case, microscopic live sessions via Deca and Penta head microscopes, television, and microscopic camera. It is the same method we are using among postgraduate students in pathology. But some aspects require further improvements, such as the attitude of other faculty, increased numbers of faculty, lack of knowledge about technology, and periodic training to use newer software [11]. Hence, the aim of the study was to develop a patient- and technology-oriented newer PBL method and compare its effectiveness with the traditional tutorial method. We also aimed to assess students’ perceptions, preferences, and performance (PPP) with respect to the newer method.

## Materials and methods

This comparative study was carried out between November 2020 and October 2021 after obtaining ethical approval from the Institutional Ethical Committee of the Nootan Medical College and Research Centre, Visnagar (IEC number: IEC/NMCRC/APPROVAL/13/2020). A total of 149 second-year MBBS students were enrolled in the study. Consent was taken from all students. The students were recruited during the second term of the second MBBS year. At this level, they have already acquired basic knowledge and have better sensitization toward the subject of pathology. We had taken prior permission to conduct this study from the institutional academic committee led by the dean and head of pathology. Before proceeding to the actual application of this newer method, departmental meetings and group discussions were held to sensitize the faculties to POPBL. Basically, in this study, we classified systems rather than students in terms of topics. We selected the main eight systems of pathology (Table 1).

**Table 1:** List of systems covered under different teaching-learning methods POPBL: Patient-Oriented Problem-Based Learning

Sr. No.	POPBL topics	Traditional lecture/tutorial topics
1	Respiratory system	Cardiovascular system
2	Gastrointestinal system	Hepatobiliary system
3	Renal system	Endocrine system
4	Male reproductive system	Female reproductive system

The first group consisted of the respiratory system, gastrointestinal system, renal system, and male genital tract. The second group comprised the cardiovascular system, hepatobiliary system, female genital tract, and endocrine system.

The first group of systems was taught under the newer method, consisting of a topics-related lecture followed by a Demonstration-Observation-Assistance-Performance (DOAP) session, which entailed POPBL with gross and microscopic features associated with the help of newer-generation audiovisual aids like the Deca and Penta head microscopes. The second group, which included the remaining four systems, was taught using older traditional methods such as lectures followed by routine gross and microscopy using a conventional binocular microscope without incorporating a problem-/case-based approach using newer-generation audiovisual aids such as the Deca and Penta head microscopes. In short, all the students in both groups were exposed to regular didactic lectures on the same topics for the understanding of the etiology and the basics of pathogenesis. The facilitators involved in traditional tutorials/lectures and POPBL classes were instructed regarding the plan and its execution.

**Figure 1:**
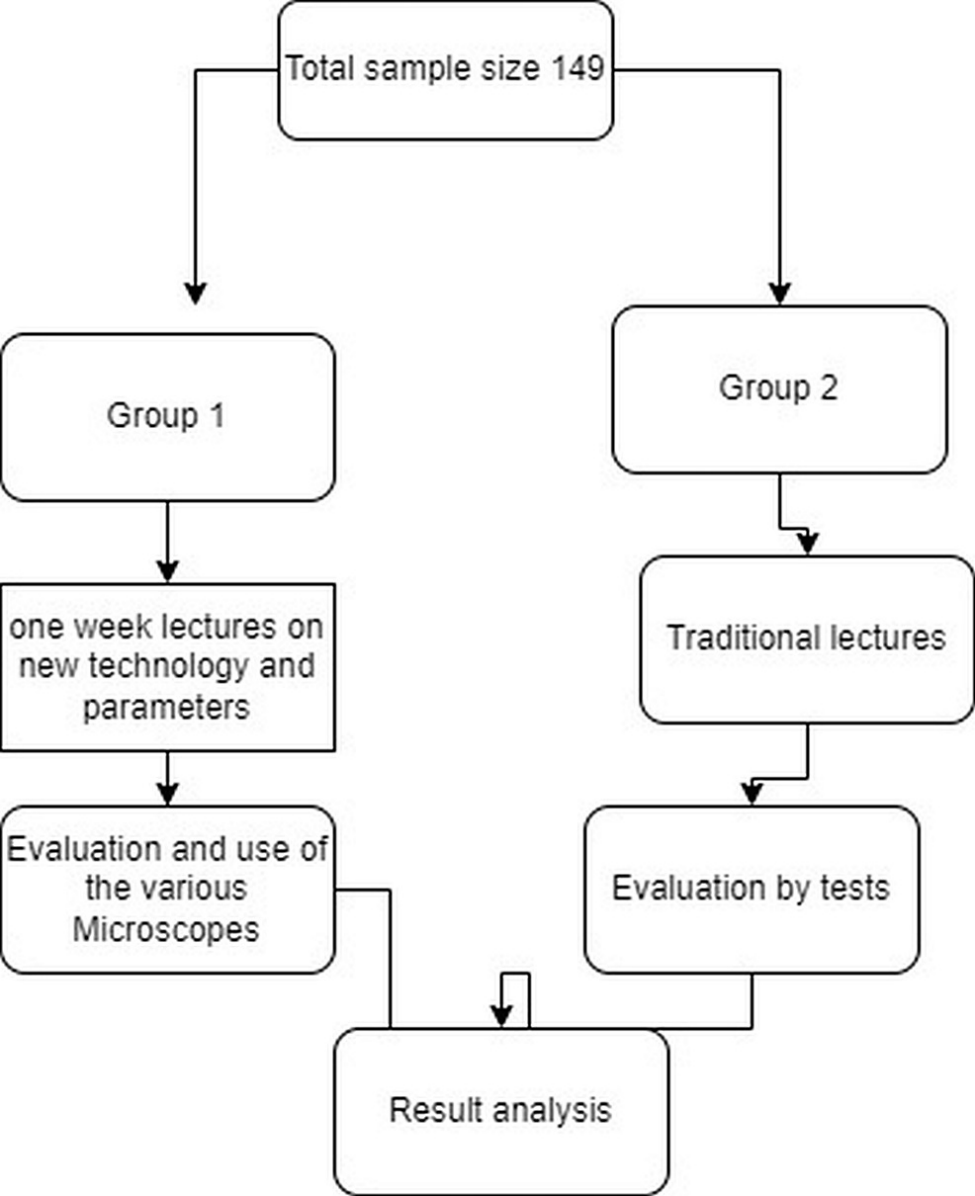
Methodology flowchart

**Figure 2:**
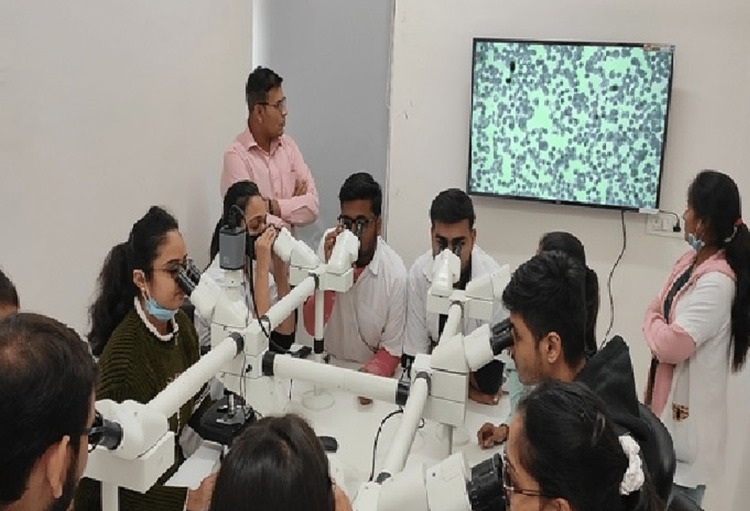
Participants using microscopes and television

We selected various groups for the POPBL session based on random matching as per students' university roll number; among the total 149 students, further categorization was done to classify students into 10 different groups, with each group consisting of 15 students. This was done because we could place more emphasis on small-group teaching and achieve a better learning curve. All these sessions were integrated into the UG teaching curriculum with proper alignment as well as horizontal and vertical integration. We structured the UG timetable so that each system had its respective theory lectures in one week, followed by DOAP sessions in the same or subsequent week, regardless of the teaching-learning method used. The purpose was to facilitate getting a detailed understanding of the etiopathogenesis and clinical correlation of a particular topic or case. In the first session, all students were given four short histories (one for each system) of patients with clinical examination data and routine laboratory findings. After one week, we initially explained the clinical scenario with basic clinical and laboratory findings to all students. Subsequently, all students went through gross specimens and microscopic slide examinations of each case with the help of newer-generation audiovisual aids like Deca and Penta head microscopes and television. All faculty members of the pathology department, such as professors, associate professors, assistant professors, and tutors, participated. Each group of students was, turn by turn, explained and demonstrated gross features of specimens and showed microscopic features of slides by expert teachers with the help of Deca and Penta head microscopes and television. Following this session, we organized another half-hour of question-and-answer sessions for students to clarify their understanding of concepts and solve their queries. Figure 1 and Figure 2 illustrate the methodology flowchart and the participants using the microscopes and the television in the study, respectively.

For group 2, which involved a traditional tutorial/lecture method session, all students attended traditional lectures followed by traditional tutorials such as viewing photographs and routine practical classes on the remaining four systems. The evaluation was done using prevalidated objective types of questions after exposure of about one week. The aim was to evaluate and compare the outcomes and students' performance between these two sets of pathology systems. Students subsequently took exams with a total of 50 marks for evaluation, which was conducted by a single examiner. There were two parts to the evaluation process: Section A (25 marks) and Section B (25 marks). A modern teaching and learning approach, POPBL, informed the questions in part A, whereas subjects addressed in classic tutorial and lecture methods informed those in section B. Statistical analyses were carried out using SPSS Statistics version 16.0 (IBM, Armonk, NY). The z-test was employed to examine the statistical significance of the differences.

## Results

We compared students' perception of POPBL with gross and microscopic features associated with the help of newer-generation audiovisual aids like Deca and Penta head microscopes and that of the traditional tutorial/lecture method (Table 2).

**Table 2:** Comparison of students' responses to POPBL and the traditional lecture/tutorial method POPBL: Patient-Oriented Problem-Based Learning

Questions	‘YES’ response of students				Z-value	P-value
	POPBL		Traditional lecture/tutorial method			
	No. of students (N=149)	%	No. of students (N=149)	%		
Do you think this type of methodology facilitates self-learning?	124	83.22	29	19.46	15.2	<0.05
Do you think this type of methodology should be used by every teacher in future classes?	132	88.59	52	34.9	13.1	<0.05
Do you think this type of methodology will help you to make a diagnosis in real clinical practice?	130	87.24	66	44.29	8.7	<0.05
Do you think self-reading before class helps in understanding class material?	116	77.85	67	44.97	5.05	<0.05
Do you think this type of methodology creates interest in the topic?	144	96.64	41	27.52	12.2	<0.05
Do you think this type of methodology helps to keep your attention in the classroom?	139	93.28	43	28.86	11	<0.05
Do you think this type of methodology is a more scientific way of teaching?	138	92.61	41	27.52	10.9	<0.05
Do you think this type of methodology strengthens students' intrinsic motivation?	125	83.89	67	44.97	6.8	<0.05
Do you think this type of methodology develops self-directed learning skills?	125	83.89	24	16.11	16.7	<0.05
Do you think this type of methodology gives a systemic approach to applying findings of cognitive psychology to the educational process?	129	86.58	35	23.49	15.1	<0.05
Do you think this type of methodology helps you in terms of better retention of the topic covered?	141	94.63	38	25.5	12.2	<0.05

Of note, when we asked students what they thought about (1) whether one method helped them learn more independently and whether it would be useful in future classes, (2) whether it would help diagnose real-world medical problems, (3) whether it would keep students engaged in class, (4) whether it was scientific in nature, and (5) whether it would help them develop self-directed learning (SDL) skills, we received a wide range of responses. With the support of current-generation audiovisual aids like Deca and Penta head microscopes, students were able to respond well to the POPBL with gross and microscopic features linked (Section 3). There was a statistically significant difference between the techniques in terms of students' responses (p=0.05) (Table 2). More than 80% of students stated that POPBL with gross and microscopic features associated with the help of newer-generation audiovisual aids like the Deca and Penta head microscopes was better than the traditional tutorial/lecture method. When students' performance in Section A of the post-session examination using the POPBL method and newer audiovisual aids like the Deca and Penta head microscopes was analyzed, it was seen that 87.92% of students scored above 75%, which was far superior to their performance in Section B using the traditional tutorial/lecture method, where only 53.02% of students scored above 75% (Table 3). This difference was statistically significant (p=0.05).

**Table 3:** Students' performance in the post-session examination POPBL: Patient-Oriented Problem-Based Learning

Marks obtained (%)	Section A (POPBL topics)		Section B (traditional lecture/tutorial topics)	
	No. of students	%	No. of students	%
100-75	131	87.92	79	53.02
50-74.99	12	8.05	54	36.24
<50	06	4.03	16	10.74
Total	149	100	149	100

Students in the POPBL session had an average attendance of 94.14%, whereas the typical lecture/tutorial technique had an attendance of 76.12% (Table 4). The difference was statistically significant (p=0.05).

**Table 4:** Comparison of students' attendance between POPBL and traditional lecture/tutorial methods POPBL: Patient-Oriented Problem-Based Learning

Session	Students' average attendance (N=149)	% attendance
POPBL method	140.27	94.14
Traditional lecture/tutorial method	113.42	76.12

## Discussion

Curriculum changes

According to several writers, medical students who have graduated often find it difficult to apply what they have learned from books [12]. About half of the physicians and residents at a major hospital were unable to conduct essential screening procedures in suspected cases of kidney disease. While they all fared well in the MCQ exam, they all had a hard time in real-world situations. Such a predicament can be attributed to a phenomenon in which individuals might acquire information but are unable to put it to use in their daily lives [12]. This is partly due to the fact that medical students in India are only taught using conventional methods. However, several researchers and educators have critiqued this approach on numerous occasions [13-16]. Recently, many medical colleges have started using PBL methods in their curriculum [5,17,18]. In the pathology department of our medical college, we adopted POPBL with gross and microscopic features associated with the help of newer-generation audiovisual aids like the Deca and Penta head microscopes to educate undergraduate students. This method had already been implemented for postgraduate students in various medical colleges in India; however, very few medical colleges have implemented this method in their undergraduate teaching curriculum. This is due to staff shortages, minimal resources, and the unavailability of Deca and Penta head microscopes in many medical colleges [19].

Klegeris and Hurren [20] conducted a pharmacology-related investigation at the UBC Okanagan campus in Kelowna, British Columbia, Canada, and their findings are superior to ours in terms of various parameters. According to the results of this research, PBL improved student learning outcomes, including their ability to comprehend and retain course information.

Reflections on the efficacy of POPBL over traditional teaching methodology

According to Roche and Abraham [21], 79% of participants stated that the PBL technique helped them learn and understand the subject better. In the present study, students gave excellent positive responses (more than 80%) regarding the facilitation of the self-learning method, help in diagnosis in real-time clinical scenarios, better understanding of the topic or case, creating more interest and having deep retention of the topic, helping maintain attention in the classroom, a scientific way of teaching, strengthening their intrinsic motivation, and developing skills in SDL (a strategy that integrates the results of cognitive psychology with the educational process).

As shown in Table 3, POPBL-covered subjects fared better than lecture or tutorial-based topics in terms of student performance. A student-centered strategy, POPBL involves active engagement from each student, which has been proven to be effective in post-session examinations. Students' attendance may be used as a trustworthy indicator of student satisfaction. Students were strongly encouraged to attend this kind of creative session regardless of whether or not they had been upgraded for it based on the newer way we used to obtain data from POPBL's multiple systems. According to research by Klegeris and Hurren, PBL sessions at the UBC Okanagan campus in Kelowna, British Columbia, Canada, had much higher attendance than regular lectures [20].

## Conclusions

Based on our findings, the POPBL approach is superior to the conventional tutorial/lecture method in terms of student-learning outcomes. Students also found POPBL more appealing than standard lectures. However, the POPBL method of teaching cannot be used to achieve all competencies with the specified goals. because each small group of 15 students in POPBL needs two instructors. Due to the enormous number of students and the insufficient number of personnel in countries like India, we are unable to adequately serve all of our students. In addition, newer-generation audiovisual aids like Deca and Penta head microscopes may be used to include POPBL together with gross and microscopic characteristics to relieve the monotony of dialectic lectures, thereby allowing students to satisfy their professional needs. It also saves time for separate gross and microscopic explanations. This newer method also increased the level of undergraduate student interest in medical education and clinical practice. Hence, this is a student-centered method that provides more motivation to students and encourages active participation.
